# Infection prevention knowledge, practice, and its associated factors among healthcare providers in primary healthcare unit of Wogdie District, Northeast Ethiopia, 2019: a cross-sectional study

**DOI:** 10.1186/s13756-020-00802-w

**Published:** 2020-08-17

**Authors:** Jemal Assefa, Gedefaw Diress Alen, Seteamlak Adane

**Affiliations:** 1 South Wollo Zonal Department, Dessie, Ethiopia; 2 Department of Public Health, College of Health Science, Woldia University, Woldia, Ethiopia

**Keywords:** Knowledge, Practice, Infection prevention, Wogdie

## Abstract

**Background:**

Adequate knowledge and safe practice of infection prevention among healthcare providers are vital to prevent nosocomial infections. Thus, this study aimed to assess the level of knowledge and practices of healthcare providers towards infection prevention and its associated factors in the health facilities of Wogdie District, Northern Ethiopia.

**Methods:**

Institution based cross-sectional study was conducted among 171 healthcare providers who were selected by a simple random sampling technique. Data were collected using interviewer-administered questionnaire. Multivariable logistic regression was performed to identify factors associated with knowledge and practice of infection prevention.

**Result:**

About 70.8 and 55.0% of healthcare providers had adequate knowledge and safe practice of infection prevention respectively. Having infection prevention guideline (AOR = 3.65, 95% CI; 1.26, 10.54), taking infection prevention training (AOR = 2.2, 95% CI; 1.01, 4.75), having five years or more work experience (AOR = 1.52:95%CI; 1.13, 4.51), and working in maternity unit (AOR = 1.67:95%CI; 1.38–5.23) were positively associated with adequate knowledge of infection prevention. The odds of safe practice were higher in participants who received infection prevention training (AOR: 2.4; 95% CI; 1.01, 4.75) but lower among healthcare providers who are working in the facility which has no continuous water supply (AOR = 0.48:95% CI; 0.21, 0.83).

**Conclusion:**

A significant proportion of healthcare providers had inadequate knowledge and unsafe practice of infection prevention. To improve healthcare worker’s knowledge of infection prevention, adequate pre-service as well as on job training should be given.

## Background

Healthcare-associated infection is defined as infections acquired in the healthcare facilities during the clinical, diagnostic and therapeutic procedure that was not present at the time of the client’s admission [1]. It is a main problem encountered in healthcare delivery services worldwide and constitutes one of the most important causes of morbidity and mortality [1–3]. A systematic review of the literature reported that the pooled prevalence of healthcare associated infection was 7.6% in high-income countries and 10.1% in low and middle-income countries [4].

In Africa including Ethiopia, the prevalence of hospital-acquired infection was significantly high (12–35%) [5–8]. However, awareness of the problem remains extremely limited because of other health priorities take precedence over infection prevention and patient safety considerations [4]. Most of the healthcare associated infections are caused by the transmission of pathogens from one patient to another, especially by healthcare workers who failed to practice infection prevention measures consistently [9–12].

In Africa, many studies have shown that significant proportion of healthcare providers had inadequate knowledge on infection prevention. Only 58% of health care workers in Ghana [13] and 70–80% in Nigeria [14, 15] had adequate knowledge about infection prevention. Similarly, past evidence in Ethiopia showed that merely 54–60% of healthcare providers had adequate knowledge about infection prevention [16–18] and only 32–55% of healthcare providers demonstrated a safe practice on infection prevention [15, 17, 19].

Studies have shown that different factors such as availability of infection prevention guidelines, training on infection prevention and availability of personal protection equipment can promote safe infection prevention practices at the hospital level [16–18, 20]. However, the factors associated with knowledge and practice of infection prevention in the primary health care unite of Ethiopia have not previously been explored.

The Federal Ministry of Health (FMoH) of Ethiopia started initiatives to protect patients and health workers from healthcare associated infection by setting infection prevention standards and guidelines. Adequate knowledge of infection prevention is crucial to implement these infection prevention standards and to improve infection prevention practice. However, very little has been published on the level of knowledge and practices towards infection prevention among health workers in Ethiopia. Besides, the majority of previous studies [17, 21–23] in Ethiopia have done at the hospital level which differs from the primary health care unit in regarding to staff profile, infrastructure, training and financial support. Therefore, this study aimed to assess the knowledge and practices towards infection prevention and its associated factors among primary healthcare providers in Wogdie district, Northern Ethiopia.

## Methods

### Study design, setting and population

Institutional-based cross-sectional study design was conducted from February to May in Wogdie district which is located in the Northern part of Ethiopia. It is 581 Km away from Addis Ababa (the capital city of the country) and 640Km from Bihar Dar (regional capital city). According to Wogdie woreda health office 2019 annual plan, the district had a total population of 155, 960 and it has one governmental primary Hospital and five health centers. All healthcare workers (Laboratory, Nurse, Health officer, and Midwifery) working in all three departments of healthcare facilities (Out-patient, In-patient and Emergency department) and providing care and have direct involvement in patient care were eligible to be included in the study. Healthcare providers who were on annual leave for longer than 2 weeks, maternity leave and who were critically ill during the study period were excluded.

### Sample size determination & sampling technique

The sample size was determined by using single population proportion formula by considering the proportion of practices on infection prevention method 87.5%, from the study conducted in Dessie Referral Hospital [24] using the assumptions: 95% confidence interval, 5% margin of error. The calculated sample size was 162 but after adding a 5% non-response rate, the final sample size became 171. A simple random sampling technique (table of random number) was used to select the representative subjects from all healthcare providers list in the Woreda Health Office.

### Variables

The outcome of interest was knowledge (adequate/inadequate) and practice (safe/unsafe) towards infection prevention. Independent variables include socio-demographic characteristics, water supply, presence of infection prevention committee, presence of infection prevention guidelines in a health facility and training on infection prevention. Knowledge of infection prevention was computed from 11 questions. The mean value was used to classify HCWs infection prevention knowledge as having adequate knowledge if the score was equal or above the mean. Respondents who scored less than the mean value of correct answers were classified as having inadequate knowledge on the infection prevention. Infection prevention practices of healthcare provides were assessed for main components of infection prevention measures like hand hygiene practices, utilization of personal protective equipment (PPE), and post-exposure prophylaxes (PEP), healthcare waste management practices, and instrument disinfection practice. Respondents were asked to indicate the frequency of use (practice) for these 7 infection prevention measures. Practice assessment questions had either three or two possible alternative responses (“Always” or “Yes”, “Sometimes” and “Never” or “No”). One point was given for each acceptable or correct practice and zero point for all other responses. Practice scores were summed up to give a total practice score for each healthcare worker. Therefore, the total score of practice questions ranging from zero (all prevention measures not practiced safely) to seven (all infection prevention measures practiced safely) were classified into two categories of response: safe practice (equal or above the mean) and unsafe practice (below the mean) [25, 26].

### Data collection techniques

Two trained BSc Nurses collected the data through face-face interview using a structured and pre-tested questionnaire which was prepared in the local language (Amharic). The tool was developed after reviewing related kinds of literature [2, 17, 18, 24, 27]. Data collectors were trained and supervised during the data collection period.

### Data quality control

To assure the data quality, data collection instruments were pre-tested on 5% of the sample on two health centers of Borena woreda. For each component, a reliability test was done. The reliability coefficient for practice and knowledge items had a Cronbach’s Alpha value of 0.832 and 0.753 respectively. The data were examined for completeness and consistency during data collection on a daily base by the supervisor.

### Data analysis

Data were entered using Epi-data version 3.1 and analyzed by SPSS version 20. Before analysis, data were cleaned and checked for outliers and missing’s. Logistic regression was done and variables with a *P*-value of less than 0.2 at bivariable logistic regression model were entered into the multivariable logistic regression model. In all cases, *p*-values of less than 0.05 were considered as statistically significant.

## Result

### Socio-demographic characteristics

In this study, a total of 171 study participants were included and the mean age of the study participants was 27.98 with SD ± 3.56 years. Of the total participants, 119 (69.6%) of them were males and the majority 110 (64.3%) were married. About 83(48.5%) study participants were Nurses regardless of their educational level. In terms of educational status, 102(59.6%) study participants were diploma and nearly one-third of respondents were currently working at the outpatient clinics (Table 1).

**Table 1:** Socio-demographic characteristic of health care providers in five health facilities of Wogdie district, South Wollo zone, Amhara region, Ethiopia, 2019

Variables	Category	Frequency	Percentage (%)
Age	18–27	84	49.1
	28–37	82	48.0
	38–47	5	2.9
Sex	Male	119	69.6
	Female	52	30.4
Marital status	Married	110	64.3
	Single	61	35.7
Profession	Nurse	68	39.8
	Midwifery	33	19.3
	Health Officer	26	15.1
	Laboratory technologist/technician	29	17.0
	Pharmacist/pharmacy-technician	15	8.8
Educational status	Degree and above	69	40.3
	Diploma	102	59.7
Work experience in health facility	Less than 5 years	114	66.7
	> = 5 years	57	33.3
Currently working unit	Outpatient department	51	29.8
	Emergency and Triage	22	12.9
	Maternity unit	33	19.3
	Inpatient clinic	13	7.5
	Laboratory	29	17.0
	Pharmacy	15	8.8
	Under-five clinic	8	4.7

More than two-thirds of health facilities had infection prevention committee and around 73% of health facilities had infection prevention guidelines. About 33(19.3%) study participants didn’t take any training on infection prevention and universal precaution (Table 2).

**Table 2:** Characteristic of health care providers in Wogdie district, South Wollo Zone, Amhara region, Ethiopia, 2019

Characteristics	Category	Frequency	Percentage (%)
Type of health facility	Rural Health center	98	57.3
	Urban health center	73	42.7
Members of infection prevention committee	Yes	119	69.5
	No	52	30.5
Access infection prevention guideline	Yes	124	72.5
	No	47	27.5
Infection prevention training	Yes	58	33.9
	No	113	66.1
Availability of continuous water supply	Yes	88	51.5
	No	83	48.5

### Knowledge of health care providers on infection prevention

In this study, 70.8% of participants had adequate knowledge about infection prevention. Sixteen (9.4%) were believed that gloves cannot provide complete protection against acquiring infection. One hundred sixty-three (95.3%) of the study participants responded that washing hands with soap or an alcohol-based antiseptic decreases the risk of transmission of hospital acquired pathogens (Table 3).

**Table 3:** Knowledge questions for health care providers in health facilities of Wogdie district, South Wollo zone, Amhara region, Ethiopia (*n* = 171), 2019

Knowledge Variables	Category	Frequency	Percentage (%)
Have you heard about infection prevention principle?	Yes	171	100
	No	0	0
Do you think that gloves cannot provide complete protection against acquiring/transmitting infection?	Yes	155	90.6
	No	16	9.4
Do you think that healthcare-associated pathogens can be found on normal and intact patient skin?	Yes	163	95.3
	No	18	4.7
Do you think that washing your hands with soap or alcohol-based antiseptic decreases the risk of transmission of hospital acquired infection?	Yes	163	95.3
	No	8	4.7
Do you think that use of an alcohol-based antiseptic for hand hygiene is as effective as soap and water if hands are not visibly dirty?	Yes	168	98.2
	No	3	1.8
Do you think that gloves reduce the contamination of the hand but do not prevent it completely?	Yes	137	80.1
	No	34	19.9
Do you think that no need to wash hands before doing procedures that do not involve bodily fluids?	Yes	33	19.3
	No	138	80.7
Do you think that no need to wear the same pair of gloves for multiple patients as long as there is no visible contamination?	Yes	130	76.0
	No	41	24
Do you think TB is carried in airborne particles that are generated from patients with active pulmonary TB?	Yes	169	98.8
	No	2	1.2
Do you know to what level safety boxes should be filled before closing and sealing?	Full	16	9.4
	¾ full	155	90.6
Do you know specific waste disposal buckets according to the level of their contamination?	Yes	167	97.7
	No	4	2.3

### Practice of health care providers on infection prevention

In this study, 55% of healthcare providers had safe infection prevention practice. From all participants, 68.4% wash their hands before patient care and 77.2% used soap to wash their hands. Around 50.3% of healthcare providers have used all personal protective equipment’s and 90.6% participant changes chlorine solution every 24 h (Table 4).

**Table 4:** Infection prevention practice questions for health care providers in health facilities of Wogdie district, South Wollo zone, Amhara region, Ethiopia (n = 171), June 2019

Variables	Category	Frequency(N)	Percentage (%)
How often you wash your hands with proper detergent after contact with patient?	Always	68	39.8
	Sometimes	87	50.9
	Never	16	9.3
Do you use antiseptic hand rub to clean hands?	Yes	160	93.6
	No	11	6.4
How often do you use all personal protective equipment’s as per standard to prevent infection?	Always	86	50.3
	Some times	85	49.7
When do you change chlorine solutions that used for instrumental processing?	Every 24 h	155	90.6
	After 2 days	16	9.4
How often do you use glove when you perform procedures that need wearing glove?	Always	142	83.0
	Some times	29	17.0
Have you ever exposed to blood or other body fluids of patients through contact or unprotected skin?	Yes	122	71.3
	No	49	28.7
What measure did you take if you are exposed to blood or fluids, needle stick injury?	Only taking Post exposure prophylaxis	19	11.1
	Only clean by alcohol	68	39.8
	Only washing with water	2	1.2
	Taking Post exposure prophylaxis and clean by alcohol	32	18.7
	Taking post exposure prophylaxis and washing with water	7	4.1
	Clean by alcohol and washing with water	14	8.2
	All action taken	29	17.0
Did you practice high-level disinfection where sterilization is not applicable?	Yes	124	72.5
	No	47	27.5
What is your facility sterilization technique?	Boiling	19	11.1
	steam sterilization	152	88.9

### Factors associated with knowledge of infection prevention

In this study, all variables with a *P*-value of less than 0.2 at bivariate logistic regression model were included in the multivariable analysis. Thus age, sex, educational status, profession, work experience, presence of infection prevention guideline, getting of infection prevention training, availability of continuous water supply, type of health center and currently working unit were evaluated as possible factors associated with knowledge of infection prevention. But, we removed two variables; type of health center and member of infection prevention committee to avoid multicollinearity (detected through chi-square test). In multivariable analysis presence of IP guidelines, IP training, work experience and currently working unit were significantly associated with knowledge of infection prevention (*P* < 0.05). The odds of having adequate knowledge of infection prevention among health care providers who have IP guidelines in their health institutions were 3.7 times higher (AOR: 3.65, 95% CI;1.26, 10.54) than those who have not IP guideline. The odds of having adequate knowledge towards infection prevention were higher in health worker who has trained about infection prevention (AOR = 2.19: 95% CI; 1.01, 4.75) than those who were not trained. Similarly, participants who had more than 5 years’ work experience had higher odds of having adequate infection prevention knowledge (AOR = 1.52: 95%CI; 1.13–4.51). Healthcare providers who were currently working in maternity and laboratory unit had higher odds of having adequate knowledge about IP ((AOR = 1.67:95%CI; 1.38–5.23), (AOR = 2.56:95%CI; 1.26–4.13)) respectively (Table 5).

**Table 5:** Bivariate and multivariate logistic regression of factors associated with knowledge of health care providers towards infection prevention of Wogdie district, south Wollo zone Amhara region, Ethiopia, 2019

Characteristics		Knowledge status		COR (95%CI)	AOR (95%CI)
		Adequate	Inadequate		
Sex	Male	80(67.2)	39(32.8)	0.55(0.25–1.18)	1.03(0.34–3.09)
	Female	41(78.8)	11(21.2)	1	1
Educational status	Diploma	65(63.7)	37(37.3)	1	1
	Degree	53(80.3)	13(19.7)	2.47(1.17–5.20)	1.05(0.32–3.44)
Work experience	< 5 Years	75(65.7)	39(34.3)	1	1
	> = 5 years	46(80.7)	11(19.3)	0.46(0.21–0.98)	1.52(1.13–4.51) *
IP Guideline	Yes	100(80.6)	24(19.4)	5.16(2.49–10.68)	3.65(1.26–10.54) *
	No	21(44.7)	26(55.3)	1	1
IP Training	Yes	32(42.1)	26(24.8)	17.64(6.87–45.33)	2.19(1.01–4.75)*
	No	44(57.9)	79(75.2)	1	1
Currently working unit	Outpatient department	30(32.2)	21(26.9)	1	
	Emergency and Triage	6(6.5)	16(20.5)	1.25(0.53–2.04)	0.25(0.13–1.54)
	Maternity unit	23(24.7)	10(12.8)	1.31(1.08–3.38)	1.67(1.38–5.23)*
	Inpatient clinic	8(8.6)	5(6.4)	0.78(0.25–2.81)	1.38(0.48–2.51)
	Laboratory	15(16.1)	14(17.9)	1.46(1.13–3.43)	2.56(1.26–4.13)*
	Pharmacy	6(6.5)	9(11.5)	0.84(0.39–3.03)	0.54(0.17–1.57)
	Under-five clinic	5(5.4)	3(3.8)	0.91(0.32–1.84)	0.87(0.57–3.47)

*IP* infection prevention, *COR* crude odds ratio, *AOR* adjusted odds ratio; * = significantly associated at *P* < 0.05

### Factors associated with infection prevention practice

In this study, age, sex, educational status, profession, work experience, presence of infection prevention guideline, getting of infection prevention training, availability of continuous water supply, type of health center and currently working unit were evaluated as possible factors associated with safe infection prevention practices. But, getting infection prevention training and the availability of continuous water supply in the health facility were significantly associated (*P* < 0.05) with safe infection prevention practices in the multivariable analysis. Healthcare providers who have trained on infection prevention were 2.2 times (AOR: 2.19, 95% CI; 1.01–4.75) more likely to have good practice than those who were not trained. The odds of safe infection prevention practices were 52% lower among healthcare providers who work in health facility which have not continuous water supply (AOR = 0.48:95% CI;0.214–0.832)) than their counterparts (Table 6).

**Table 6:** Bivariable and multivariable logistic regression of factors associated with infection prevention practice of health care providers in Wogdie district, south Wollo zone, Amhara region, Ethiopia 2019

Characteristics		Infection prevention practice status		COR 95% CI	AOR 95% CI
		Safe	Unsafe		
Sex	Male	80(67.2)	39(32.8)	0.55(0.26–1.19)	1.21(0.52–2.80)
	Female	41(78.8)	11(21.2)	1	1
Educational status	Degree	53(80.3)	13(19.7)	2.47(1.17–5.20)	0.78(0.32–1.91)
	Diploma	65(63.7)	37(37.3)	1	1
Work experience	< 5 Years	75(65.7)	39(34.3)	0.46(0.21–0.99)	0.89(0.42–1.913)
	> = 5 years	46(80.7)	11(19.3)	1	1
Presence IP guideline	Yes	100(80.6)	24(19.4)	5.16(2.49–10.68)	0.95(0.83–4.55)
	No	21(44.7)	26(55.3)	1	1
Infection prevention training	Yes	114(82.6)	24(17.4)	17.64(6.87–45.33)	2.19(1.13–4.75) *
	No	7(21.2)	26(78.8)	1	1
Availability of continuous water supply	Yes	74(52.1)	14(48.3)	1	1
	No	68(47.9)	15(51.7)	0.86(0.34–0.95)	0.48(0.21–0.83) ^*^
Currently working unit	Outpatient department	39(36.1)	12(22.6)	1	1
	Emergency and Triage	9(8.3)	13(24.5)	0.21(0.13–1.24)	0.25(0.13–1.54)
	Maternity unit	18(16.7)	5(9.4)	1.11(0.79–1.21)	1.67(0.89–3.61)
	Inpatient clinic	7(6.5)	6(11.3)	0.28(0.15–1.63)	0.68(0.34–1.97)
	Laboratory	22(20.4)	7(13.2)	0.97(0.53–2.76)	3.24(1.68–6.27) ^*^
	Pharmacy	7(6.5)	8(15.1)	0.84(0.39–3.03)	0.54(0.17–1.57)
	Under-five clinic	6(5.6)	2(3.8)	0.47(0.21–1.35)	0.79(0.36–2.17)

* = significantly associated at *P*-value < 0.05

## Discussion

Adequate knowledge about infection prevention is vital in reducing healthcare-associated infection. The finding in this study suggested that approximately one-third of healthcare providers working in primary healthcare unit demonstrated inadequate knowledge about infection prevention. This finding is supported by previous studies [14, 16, 28] done in different parts of Africa. But it was low when compared with studies done in Ethiopia [17, 23]. This discrepancy may be due to differences in the study settings. The majority of previous studies were done at teaching and referral hospitals but this study was limited to the primary healthcare units (health centers). In teaching and referral hospitals healthcare providers might get the opportunities for various infection prevention training which could increase knowledge level about infection prevention [14, 29].

Even though FMoH of Ethiopia is scaling-up its activities related with infection prevention, the current study showed that only 55% of healthcare providers had safe infection prevention practices. This finding was in line with a study done in Bahir-Dar [23] but lower when compared with studies done in other parts of Ethiopia [24, 30]. The discrepancy might be due to a difference in the study setting and composition of healthcare providers [24]. In Ethiopia, the composition of health worker staffs were different among hospitals and primary healthcare unit. Majority of health workers in hospitals were experienced and specialized which might improve their practice towards infection prevention. This finding implies that improving safe infection prevention practices are vital in primary healthcare units of Ethiopia where a high proportion of patients developed healthcare associated infection [31].

Remarkably, in the current study, about 90% of health care providers believed that gloves can’t provide complete protection against acquiring infection. This finding is higher than previous studies done in Ethiopia [17, 18] and Eritrea [32]. As similar to previous studies done in Ethiopia [17, 27], in this study, only one-third of healthcare providers had hand hygiene practice after patient care and 68.4% of healthcare workers wash their hands before patient care.

This study revealed that healthcare providers who had more than five years of work experience were 1.5 times more likely to have adequate knowledge than their counterparts. This is in line with findings from Ethiopia [17, 25]. This might be due to the fact that as the number of years of practice increases, healthcare providers are exposed to infection prevention information and became more experienced through working with senior staff.

According to WHO recommendation, all primary healthcare facilities should develop their own standard operating procedures based on national infection prevention and control guidelines [33, 34]. Developing and implementation of infection prevention guidelines is one of the core components of infection prevention and control programs at all healthcare facilities [33, 34]. In this study, similar to previous studies [18, 22], the presence of infection prevention guidelines in health institutions also increases the odds of having adequate knowledge about infection prevention. This might be attributed to the fact that healthcare workers who have infection prevention guidelines were more likely to get updated information, which improves their knowledge of infection prevention.

Healthcare providers who are currently working in maternity and laboratory unit had higher odds of having adequate knowledge about IP. This might be due to the availability of infection prevention standard operating procedures in the laboratory unit which might improve their knowledge. Besides, this could be due to the difference in infection prevention training among units.

Health workers who had ever attended any form of training program on infection prevention were more likely to practice than those who didn’t take training on infection prevention. This is supported by a study done in Ethiopia [18]. According to different international guidelines, infection prevention education & training is the core components of infection prevention and control programs [33, 34]. Infection prevention and control guidelines in combination with health care workers’ education and training are effective to reduce hospital acquired infection [34]. Previous studies have verified that training of health care workers on infection control has a valuable effect on healthcare staff by improving their compliance with the standard precaution [35, 36]. But the current study showed that only 33.9% of the health workers had ever attended any form of training program on infection prevention. This implies more than two-third of healthcare providers need training for infection prevention. Similarly, healthcare providers who had infection prevention guidelines were more likely to have safe infection prevention practice than those who had no infection prevention guidelines. The finding highlights the necessity of infection prevention guideline in the improvement of healthcare providers’ practice.

Healthcare providers working in health facilities without continuous running water supply were 52% less likely to have safe infection prevention practices as compared with HCPs working in a facility with continuous water supply. This finding is supported by a study done in Ethiopia [30].

One of the limitation of this study was temporal relationships cannot be established between the explanatory and outcome variables due to the cross-sectional nature of study design. Recall bias might be the other limitation of this study. Finally, since the study was conducted in governmental healthcare facilities, generalizability of the study findings is limited to these governmental healthcare facilities.

## Conclusion

The findings of this study showed that a significant proportion of HCPs had no adequate knowledge on infection prevention and 45% of healthcare providers practiced infection prevention unsafely. Knowledge towards infection prevention has been significantly associated with work experience, getting of infection prevention training and presence of infection prevention guideline. As a result, to improve knowledge on infection prevention, adequate pre-service as well as on job training for HCPs should be given. Besides, infection prevention guideline should be availed to improve knowledge and practice of infection prevention.

## Data Availability

The data can be available from corresponding author.

## Abbreviations

AOR: Adjusted Odds Ratio
COR: Crude Odds Ratio
HCP: Health Care provider
IP: Infection Prevention
IPC: Infection Prevention Control
WHO: World Health Organization
