# Correction: Infection prevention knowledge, practice, and its associated factors among healthcare providers in primary healthcare unit of Wogdie District, Northeast Ethiopia, 2019: a cross-sectional study

**DOI:** 10.1186/s13756-023-01298-w

**Published:** 2023-09-29

**Authors:** Jemal Assefa, Gedefaw Diress Alen, Seteamlak Adane

**Affiliations:** 1 South Wollo Zonal Department, Dessie, Ethiopia; 2 Department of Public Health, College of Health Science, Woldia University, Woldia, Ethiopia

**Antimicrobial Resistance & Infection Control (2020) 9:136**

10.1186/s13756-020-00802-w

The original article [1] contained an error in author, Gedefaw Diress Alen ’s name which has since been amended.

